# Are outpatient transperineal prostate biopsies without antibiotic prophylaxis equivalent to standard transrectal biopsies for patient safety and cancer detection rates?A retrospective cohort study in 222 patients

**DOI:** 10.1186/s13037-021-00303-8

**Published:** 2021-08-21

**Authors:** Majdee Islam, Rodrigo Donalisio Da Silva, Alan Quach, Diedra Gustafson, Leticia Nogueira, Nathan Clark, Fernando J. Kim

**Affiliations:** Department of Surgery / Division of Urology, Denver Health Medical Center, 777 Bannock St, Denver, Pavilion A, 3RD Floor, Surgery Administration, Denver, CO 80204 USA

**Keywords:** Prostate cancer, Transperineal biopsy, Transrectal, Infection, Complications

## Abstract

**Background:**

To describe our experience with outpatient transperineal biopsy (TPB) without antibiotics compared to transrectal biopsy (TRB) with antibiotics and bowel preparation. The literature elicits comparable cancer detection, time, and cost between the two. As antibiotic resistance increases, antimicrobial stewardship is imperative.

**Methods:**

In our retrospective review, we compared the TPB to TRB in our institution for outpatient prostate biopsies with local anesthesia from June 1st, 2017 to June 1st, 2019. Patients had negative urinalysis on day of procedure. Patients presenting with symptoms concerning for UTI followed by positive urine culture were determined to have a UTI.

**Results:**

Two hundred twenty-two patients met inclusion criteria. Age, race, BMI, pre-procedure PSA, history of UTI, BPH or other GU history were similar between both groups. Two TPB patients (1.8%) had post-procedure UTI; one received oral antibiotics and one received a dose of intravenous and subsequent oral antibiotics. There were no sepsis events or admissions. Six TRB patients (5.4%) had post-procedure UTI; five received oral antibiotics, and one received intravenous antibiotics and required admission for sepsis. One TPB patient (0.9%) had post-procedure retention and required catheterization, while four TRB patients (3.6%) had retention requiring catheterization. No significant difference noted in cancer detection between the two groups.

**Conclusion:**

Outpatient TPB without antibiotic prophylaxis/bowel prep is comparable to TRB in regard to safety and cancer detection. TPB without antibiotics had a lower infection and retention rate than TRB with antibiotics. Efforts to reduce antibiotic resistance should be implemented into daily practice. Future multi-institutional studies can provide further evidence for guideline changes.

**Supplementary Information:**

The online version contains supplementary material available at 10.1186/s13037-021-00303-8.

## Introduction

Prostate cancer accounts for almost one in five new cancer diagnoses [1]. In the last couple decades, there has been significant advances in early prostate cancer detection [2, 3]. In recent years detection and diagnosis has become more controversial, and a significant part of this comes from diagnosis with tissue biopsy of prostate cancer. Conventional systematic sextant transrectal prostate biopsy (TRB) with transrectal ultrasound was first reported in the late 1980s and has been the gold standard since then [4, 5]. However, transrectal biopsy complications include fever, infection, sepsis, urinary retention, and rectal bleeding, among others [6, 7]. Transperineal biopsy (TPB), an alternative option to TRB for outpatient prostate biopsy, is not widely used in the outpatient setting, especially without MRI-guidance or with saturation, particularly in the US [8]. This is likely due to previous freehand techniques being difficult to perform outpatient until recent FDA clearance of a single access needle with PrecisionPoint™ (Perineologic) [9]. The literature elicits cancer detection, prediction of final cancer laterality, procedure time, and cost of outpatient procedure itself being comparable between TPB and TRB [7, 8, 10]. Due to rectal flora resistance and overall antibiotic resistance, it becomes imperative to reduce antimicrobial use, especially with costs of post-biopsy infectious complications estimated up to $623 million annually [7, 11]. To our knowledge, there is no significant data on TPB performed without pre-procedure antibiotics compared to TRB performed in the United States.

## Materials and methods

We analyzed our experience with TPB completed without antibiotics compared to TRB with antibiotic prophylaxis and bowel preparation. After institutional review board approval, we performed a retrospective review via electronic medical record at our institution of all outpatient prostate biopsies performed with local anesthesia between June 1st, 2017 and June 1st, 2019.

TPB technique encompassed the patient being placed in the lithotomy position with chloraprep™ to the perineum for preparation. Then biopsy performed with use of PrecisionPoint™ device which includes a rail/clamp assembly that clamps onto our rectal BK8848 US probe with a 15 g access needle**.** A total of 30 cc of 1% of Lidocaine using a spinal needle is injected in the perineum raising a wheal at the skin prior to placing the access needle into the skin, and more local anesthetic is injected into the pelvic floor adjacent to the prostatic apex under transrectal ultrasound guidance on each side of the prostate. (See Additional file 1) The prostate is then measured in standard fashion.

Twelve total biopsy cores were taken, 6 on each side with 2 anterior, 2 posterolateral, and 2 posteromedial biopsies taken on each side. (See Additional file 2).

Standard TRB technique was performed with pre-procedural AUA guideline-based antibiotics and bowel preparation. Patient placed in left lateral decubitus position. Anesthetic was then performed. Periprostatic nerve block group; 5 cc of 2% lidocaine was separately injected between prostate base and seminal vesicle – the region where both neurovascular bundles are found utilizing a total of 30 cc of 1% lidocaine on each side of the prostate in particular near the apex. Prostate was then measured in standard fashion. A BK8848 probe with biplane guide was used, and 12 biopsies were performed with 6 on each size including two at the base, two mid, and two apical biopsies.


**Additional file 1.** Local anesthetic prior to prostate biopsy. In this video, we demonstrate our technqiue for local anesthesia in patients undergoing transperineal prostate biopsy.



**Additional file 2.** Prostate Biopsy Core. In this video, we demonstrate our technique for obtaining 12 cores in our transperineal prostate biopsy.


Urinary tract infection was defined as the presentation of symptoms (irritative voiding, hematuria, foul-smelling urine, and suprapubic pain) within 1 month of biopsy that triggers a urine culture order that proved to be positive. Patients that continued to be asymptomatic after biopsy were further not tested. Sepsis was defined as meeting systemic inflammatory response syndrome (SIRS) criteria with a possible focus of infection. SIRS criteria was defined as two or more of the following: temperature > 38 °C or < 36 °C, heart rate > 90/min, respiratory rate > 20/min or PaCO_2_ < 32 mmHg (4.3 kPa), white blood cell count > 12,000/mm^3^ or < 4000/mm^3^ or > 10% immature bands. Urinary retention was defined as a patient who returned to clinic or an urgent care/emergency room who required urinary catheterization within 2 weeks of the procedure. History of urinary tract infection was defined as having a positive urine culture in the previous year before the biopsy.

### Data analysis

Demographics, patient history, risk factors, and post-procedural complications/outcomes were abstracted. The patients were then divided into groups of perineal biopsy versus rectal biopsy. Differences between the two groups was compared using two-sided t-tests for quantitative data, and chi square or Mann-Whitney U test for qualitative data. *P* < 0.05 was considered significant.

### Inclusion criteria

Patients who underwent transperineal biopsy at our institution without any antibiotics, and an equal number of our most recent transrectal biopsy patients who received AUA guideline-based antibiotics and bowel preparation prior to the procedure, were included. All patients had a negative urinalysis on day of procedure. We excluded any patients with an indwelling catheter up to a month prior, patients with less than 2 months of follow up post procedure, and patients whose procedure was performed less than 2 months prior to review. For TPB patients, we also excluded those who received antibiotics. For TRB patients, we excluded those who did not receive antibiotics or a bowel preparation prior to biopsy.

After reviewing all our TPB patients retrospectively and excluding those who did not meet inclusion criteria, we had 111 TPB patients. We then retrospectively reviewed an equal number of our most recent TRB patient who met inclusion criteria.

## Results

A total of 222 patients met inclusion criteria. History of cancer was statistically significantly, though this was likely due to some transperineal patients having repeat and/or surveillance biopsies with known history of cancer (Table 1).

**Table 1:** Patient Demographics and Risk Factors

	All pts (average or %)	Perineal (only no foley or abx) – avg/% ***n*** = 111	Rectal (only no foley) -avg/% *n* = 111	***P*** value (if applicable)
**Median Age (ranges)**	**63 (43–76)**	**63 (43–76)**	**63 (50–79)**	**0.0690**
**Race**				
**Caucasian**	**87 (39%)**	**51 (46%)**	**36 (32%)**	**0.0392**
**African American**	**54 (24%)**	**21 (19%)**	**33 (30%)**	**0.0605**
**Hispanic**	**65 (29%)**	**34 (31%)**	**31 (28%)**	**0.6581**
**Unknown/Other**	**16 (7%)**	**5 (4.5%)**	**11 (10%)**	**0.1194**
**Median BMI (range)**	**26.4 (11.35–49.32)**	**26.2 (18.44–41.85)**	**26.7 (11.35–49.32)**	**0.6182**
**Prior GU hx?**				
**BPH/LUTS**	**134 (60.3%)**	**66 (59.4%)**	**68 (61.3%)**	**0.7837**
**Previous Cancer**	**28 (12.6%)**	**21 (18.9%)**	**7 (6.3%)**	**0.0047**
**Other**	**16 7.2(%)**	**10 (9%)**	**6 (5.4%)**	**0.299**
**Indwelling catheter?**	**0 (0%)**	**0 (0%)**	**0 (0%)**	**N/A**
**Prior UTI?**	**17 (7.7%)**	**8 (7.2%)**	**9 (8.1%)**	**0.8007**
**Pre-procedure PSA- Median**	**7.69 (1.05–2102.48)**	**7.69 (1.05–471)**	**7.66 (1.23–2102.48)**	**0.2344**

With regards to complications, two TPB patients had post procedure UTI; one received oral antibiotics and one received a dose of intravenous antibiotics and subsequent oral antibiotics. Neither required hospital admission. There were no sepsis events. Six TRB patients had post procedure UTI; five received oral antibiotics, and one received intravenous antibiotics and required hospital admission due to sepsis.

None of the patients with retention required admission.

There was no statistically significant difference found between the TPB and TRB cohorts in cancer detection overall or in cancer detection with low, intermediate, and high-risk disease (Table 2)

**Table 2:** Complications and Cancer Detection Rates

	Perineal (n = 111)	Rectal (n = 111)	***P*** value
**UTI after biopsy**	**2 (1.8%)**	**6 (5.4%)**	**0.280**
**Bacteria and resistances**	**- Contam skin flora** **- Citrobacter freundii; penicillin resistant.**	**- Gram negative bacilli; neg follow up culture** **-Klebsiella pneumonia, ampicillin resistant** **-Enterococcus resistant to tetracycline** **- Enterococcus without sensitivities** **- UCx with ‘3 isolates’ but symptomatic** **- Coag negative staph, resistant to bactrim**	
**Urinary retention after biopsy?**	**1 (0.9%)**	**4 (3.6%)**	**0.369**
**Cancer Detected**	**59 (53.1%)**	**50 (45%)**	**0.227**
**Low Risk**	**16 (27.1%)**	**14 (28%)**	**0.918**
**Int Risk**	**26 (44%)**	**15 (30%)**	**0.131**
**High Risk**	**17 (28.8%)**	**21 (42%)**	**0.150**

.

## Discussion

Prostate biopsy is integral to prostate cancer diagnosis and treatment. Per the AUA and NCCN guidelines, management is based on PSA, patient status, and data from biopsy. Many men that seek urology care will require a prostate biopsy during their lifetime and associated complications are not negligible. This include but are not limited to fever, infection, sepsis, urinary retention, and rectal bleeding, and includes up to 4–5% hospital admissions [6, 7, 12].

There has been a recognized increase in the prevalence of antibiotic resistant organisms, resistance in rectal flora with ESBL bacteria and quinolone-resistant bacteria and transrectal prostate biopsy sepsis [7, 12–14]. Consequently, targeted antibiotics to prebiopsy rectal swabs, or additional antibiotics on top of the AUA recommended ones have been used. However, this often cause more resistant bacteria to arise [7, 12–14]. This was seen in our institution; *E. coli* resistance to Levofloxacin was found to be 15% and to Bactrim was 26%. Our antibiotic stewardship team recommended that we switch to Fosfomycin. However, we noted this costed $2104/daily dosing. This is compared to Bactrim and Levaquin at $10/daily dosing. After contemplation we then made the decision to start using outpatient transperineal biopsies without antibiotics and compared them to transperineal biopsies with a focus on the infectious risks between them. The rationale for removing the antibiotic prophylaxis is that the TPB needle does not cross the rectum. Instead, the needle goes through 1 prepared skin entry and urinary tract was screened for infection immediately prior to the procedure.

It is important to note that nearly all studies, including comparative and meta-analyses papers, originate internationally where many studies are based on biopsies performed with MRI fusion targeted biopsies and saturation biopsies in the operating room with most cases using general anesthesia. Although performed transperineally, these used a brachytherapy template or freehand, and therefore likely differed in length of time and in methodology.

### Infection and retention

Our data comparing TPB without antibiotic prophylaxis and TRB showed a lower complication rate and lower post-biopsy infection rate with TPB. Moreover, we did not have any post-biopsy admissions for sepsis in TPB. We had multiple admissions with our transrectal biopsy cohort, including a multi-day ICU admission for IV antibiotics due to urosepsis.

Similar outcomes have also been reported. A meta-analysis performed by Xiang et al. found in studies comparing transperineal and transrectal biopsies (some including MRI fusion-targeted and saturation biopsies) that the transperineal approach decreased the risk of rectal bleeding and fever [8]. Grummet et al. published in 245 transperineal biopsies (all performed under general anesthesia with a brachytherapy grid) that they had no readmissions for infection [7]. Pepe et al. noted in their cohort of patients who underwent freehand transperineal biopsy, the post-procedure UTI rate was 0.7%. None had sepsis. However, in this study Pepe et al. did administer antibiotic prophylaxis, and many of their biopsies were saturation biopsies which were found to have more a higher rate of complications than standard 12 core biopsies [15]. In a single center retrospective study using outpatient transperineal biopsy, Meyer et al. saw no post-biopsy infections in their 43 patient non-comparative study [9].

On the other hand, with transrectal biopsies, the published post-biopsy infection rate is as high as 7%, with sepsis rates up to 3.6% [7, 14].

These findings should not be surprising, as during transrectal biopsies the biopsy needle passes from rectal mucosa into the prostate which in principle violates a potentially sterile area. This results in rectal flora being introduced into the prostate, which as we know is a very vascular gland and in effect increases a significant risk of bloodstream invasion of the rectal flora [7]. However, with transperineal biopsy Thompson et al. reports there are lower rates of plasma endotoxin and bacteremia with only skin flora found with bacteremia, theoretically giving then a lower risk of infection/sepsis [16].

With urinary retention as a known complication of prostate biopsy, we evaluated post-biopsy retention rates as well. Our study also showed transperineal biopsy had a lower rate of urinary retention post-biopsy than transrectal biopsy, at 0.9 and 3.6%, respectively. In comparison, Meyer et al. found a retention rate of 4.7% in their 43 patient transperineal biopsy experience [9]. In addition, Huang et al. reported a 3% rate of retention with TPB compared to a 12% rate after TRB, with risk factors being large prostate volume, bulging prostate transitional zone and high IPSS scores [17]. Moreover, in both meta-analyses by Xiang et al. and Shen et al., the retention rate after TPB was found to be to be similar to TRB – and this was including MRI fusion-targeted biopsies and saturation biopsies that were not performed outpatient [8, 18].

### Cancer detection

Another discussion point is the efficacy of transperineal compared to transrectal biopsy in detection of prostate cancer. There have been numerous studies internationally, one of the larger ones including a study by Hara et al., as well as the meta-analyses by Xiang et al. and Shen et al. eliciting that the two modalities were similar in cancer detection and diagnostic efficiency [8, 18, 19].

It has also been reported that the transperineal approach was better at detecting tumors in the apex and transitional prostate zones [20, 21], though this is refuted in other studies that note there is no difference in peripheral, transitional, or apex detections between the two approaches [18]. Historically, it is difficult to access the anterior zone of the prostate in particular; in this location, tumors are found at higher grades and stages, and improved ability has been noted to access this area with the perineal approach [9, 22].

In our study, detection of prostate cancer by TPB is similarly efficacious as compared to that reported by multiple studies including those by Meyer et al., Hara et al. and Xiang et al. [8, 11, 19]. Due to the planes of the TRB and similarities with cognitive fusion MRI, we found anecdotally that using cognitive fusion MRI for targeted biopsy was much easier to target accurately than standard TRB due to the field of view we are able to appreciate with TPB.

### Patient satisfaction

Xiang et al. found in their meta-analysis that patient pain was found to be increased with the transperineal approach; though many studies included in the meta-analysis had patients undergoing longer procedures with MRI fusion targeted and saturation biopsies [8]. However, Bass et al. noted in their study that approximately 90% of men were “not dissatisfied” after outpatient transperineal biopsy and would recommend it [23]. Furthermore, Merrick et al. and Smith et al. assessed pain with “visual analog scales”, taking scores from 0 to 10 for men undergoing transperineal biopsy, with 10 being “the worst pain imaginable”. Merrick et al. and Smith et al. found the worst scores were with associated with the local anesthetic injection, recorded at (4.2 ± 1.8), and (3.29 ± 1.64), respectively, with the rest of the procedures scores being significantly less than this and thus concluding that the patient satisfaction was appropriate [24, 25]. In our series, very few patients were unable to undergo the procedure; however, we did not measure patient satisfaction.

### Cost

The cost of a TPB and TRB are similar regarding outpatient necessities and instruments; the PrecisionPoint™ device for TPB costs $178, compared to the biplane guide for TRB at $22.25. Otherwise, both require similar local anesthesia, transrectal US, and a biopsy. However, by reducing infection rates our healthcare costs are reduced substantially. Evan et al. performed a cost analysis of post-biopsy infection admissions in a database of insurance claims between 2005 and 2012 and found the mean total payment for duration of each patient hospitalization for infection was $14,498.96 [11]. In addition, they reported that post-biopsy infection complications were found to cost $115 million in the 8-year time period analyzed, averaging to $14,000,000/year; when this was extrapolated to the entire male Medicare population, the estimation was at 623 million dollars annually [11]. In an attempt to remedy this, Taylor et al. used a rectal swab culture-directed antibiotic prophylaxis approach for their biopsies which resulted in a cost reduction of $4500 per avoided infection alone [26].

In our study, using transperineal biopsies could elicit significant decrease in infection along with decrease in antimicrobial resistance due to reduced needs for antibiotic prophylaxis, lowered infection rates, and hospital admissions for post procedure infection. After appreciating the numbers presented above, with each avoided infection and hospitalization, the cost reduction in healthcare resources related to transperineal biopsy could be drastic.

### Limitations

Limitation of our study included the retrospective nature and lack of randomization. Additionally, our study does currently have a small sample size and is at a single institution. However, our results do have promising data towards the applicability of transperineal biopsy without antibiotic prophylaxis in mainstream prostate cancer management.

## Conclusion

Outpatient TPB without antibiotic prophylaxis or bowel prep is comparable to TRB with regard to safety and cancer detection. TPB without antibiotics had a lower infection and retention rate than TRB with antibiotics. TPB offers a potential decrease in the cost of post-biopsy infection with antibiotic use and hospitalizations is substantial, and with antibiotic resistance continuing to rise, efforts to reduce antimicrobial use should be implemented into daily practice. Our study shows that TPB is safe without antibiotics, though more larger scale studies with the transperineal approach, further future cost analysis, and further patient satisfaction analyses are required to provide evidence for a change in guidelines.

## Data Availability

The datasets during and/or analysed during the current study available from the corresponding author on reasonable request.
